# Genotypic study of *Chlamydia trachomatis* for lymphogranuloma venereum diagnosis in rectal specimens from men who have sex with men: a cost-effectiveness analysis

**DOI:** 10.1186/s12879-024-09185-4

**Published:** 2024-03-07

**Authors:** David Sánchez, Josep Ferrer, Estela Giménez, Ignacio Torres, Diego Carretero, María Jesús Alcaraz, María Jesús Castaño, David Navarro, Eliseo Albert

**Affiliations:** 1 Microbiology Service, Hospital Clínico Universitario, INCLIVA Research institute, Valencia, Spain; 2 Department of Microbiology, School of Medicine, University of Valencia, Valencia, Spain

**Keywords:** Lymphogranuloma Venereum, Cost-effectiveness analysis, Chlamydia trachomatis, Economic evaluation

## Abstract

**Purpose:**

The significant proportion of asymptomatic patients and the scarcity of genotypic analysis of lymphogranuloma venereum (LGV), mainly among men who have sex with men (MSM), triggers a high incidence of underdiagnosed patients, highlighting the importance of determining the most appropriate strategy for LGV diagnosis, at both clinical and economical levels.

**Materials and methods:**

We conducted L1-L3 serovar detection by molecular biology in stored Chlamydia trachomatis-positive samples from MSM patients with HIV, another STI or belonging to a Pre-exposure prophylaxis program, to make a cost effectiveness study of four diagnostic strategies with a clinical, molecular, or mixed approach.

**Results:**

A total of 85 exudates were analyzed: 35urethral (31 symptomatic/4 positive) and 50 rectal (22 symptomatic/25 positive), 70/85 belonging to MSM with associated risk factors. The average cost per patient was €77.09 and €159.55 for clinical (Strategy I) and molecular (Strategy IV) strategies respectively. For molecular diagnosis by genotyping of all rectal exudate samples previously positive for CT (Strategy II), the cost was €123.84. For molecular diagnosis by genotyping of rectal and/or urethral exudate samples from all symptomatic patients (proctitis or urethritis) with a previous positive result for CT (Strategy III), the cost was €129.39. The effectiveness ratios were 0.80, 0.95, 0.91, and 1.00 for each strategy respectively. The smallest ICER was €311.67 for Strategy II compared to Strategy I.

**Conclusions:**

With 30% asymptomatic patients, the most cost-effective strategy was based on genotyping all rectal exudates. With less restrictive selection criteria, thus increasing the number of patients with negative results, the most sensitive strategies tend to be the most cost-effective, but with a high incremental cost-effectiveness ratio.

**Supplementary Information:**

The online version contains supplementary material available at 10.1186/s12879-024-09185-4.

## Introduction

*Chlamydia trachomatis* (CT) infection is the most frequent sexually transmitted bacterial infection (STI) in developed countries and the one with the highest incidence in Spain among under 25-year-olds [1, 2]. Among other pathologies, CT causes non-gonococcal urethritis, pelvic inflammatory disease, and tubal infertility. Lymphogranuloma venereum (LGV) is predominantly caused by invasive genotypes L1–L3, with molecular biology technologies as the main diagnostic tool [3, 4].

LGV has been considered endemic in Europe since 2003 in men who have sex with men (MSM), with an annual incidence of 1.5 cases per 100,000 population; approximately 70% of those with available serological data are seropositive [5]. CT L1–L3 is among the main pathogens of anorectal sexual transmission, alongside gonococcus, syphilis and HSV [6].

Diagnostic algorithms have traditionally recommended CT genotyping by PCR in CT-positive patients only if LGV-associated symptomatology is present [7]. However, multicenter studies have highlighted the existence of a 27% ratio of asymptomatic patients with LGV, mainly in HIV-positive MSM [8]. Taking these data into account and given the high prevalence of STI among MSM using the pre-exposure prophylaxis program (PrEP) [9], global screening for L1–L3 genotypes could be advisable in these two high-risk groups. However, there is no consensus as to the optimal approach for detecting this pathology or for prior selection of asymptomatic patients, or the risk factors to be considered [6, 10, 11].

CT genotyping results are reported by eleven Autonomous Communities in Spain, of which only seven reported positive cases. A total of 453 positive cases were recorded in 2019, 98% of whom were male [12]. These data show that LGV has a high incidence in Spain, particularly among MSM, and is probably underdiagnosed just as highlights the lack of a unified strategy for detecting genotypes related to lymphogranuloma venereum in patients infected by Chlamydia trachomatis within the country. In view of the above, we carried out a retrospective analysis by CT L1-L3 biovar detection and an economic evaluation to pinpoint the population eligible for these genotypic studies, the real incidence of LGV in our healthcare area, the costs associated with each diagnostic process and the best diagnostic strategy for our setting from a healthcare perspective.

## Materials and methods

### Patients and samples

L1-L3 biovar detection by molecular biology was carried out retrospectively on all PCR CT-positive urethral and rectal samples of at-risk patients analyzed in the Hospital Clínico Microbiology Service during routine procedures between 2018 and 2023. All samples, CT positive by PCR, had been cryopreserved to -80ºC. Samples from at-risk patients were those corresponding to the following groups: MSM HIV seropositive, MSM PreP-users, or MSM co-infected with *Neisseria gonorrhoeae*. A control group (18% of the total sample size) without other risk factors was also included.

### Analytic methods

We differentiated A–K from L1–L3 CT biovares by multiplex PCR and subsequent hybridization with specific probes in urethral or rectal exudate samples.

Gene extraction and purification was performed using the QIASymphony platform (QIAGEN, Venlo, The Netherlands) with the DSP DNA Pathogen kit. CT DNA detection was performed using the Allplex™ II STI-7 Detection kit (Seegene Inc, South Korea) on the AriaMX Real-time PCR platform, and the subsequent genotypic characterization using the STD Direct Flow Chip amplification assay (Vitro, Granada, Spain), in all cases following the manufacturer’s instructions.

### Economic evaluation

We carried out an economic evaluation using the decision tree approach to calculate the incremental cost-effectiveness ratio (ICER) of four hypothetical diagnostic strategies following the JBI critical appraisal checklist for economic evaluation recommendations [13]. These strategies were as follows: 1.- Clinical diagnosis of LGV based on the macroscopic rectal lesions characteristic of the pathology. 2.- Molecular diagnosis by genotyping of all rectal exudate samples previously positive for CT. 3.- Molecular diagnosis by genotyping of rectal and/or urethral exudate samples from all symptomatic patients (proctitis or urethritis) with a previous positive result for CT. 4.- Molecular diagnosis by genotyping of rectal and/or urethral exudate samples from all patients with a positive result for CT regardless of symptomatology. The proportion of true positives, false positives, true negatives, and false negatives for each strategy evaluated was obtained by comparing the hypothetical result we had obtained with each strategy and the real value obtained by the molecular procedure, considering the latter as the reference standard. Clinical data were obtained by clinical chart review.

To select the most cost-effective diagnostic strategy, the different strategies were ranked in order of increasing cost. Strategies dominated by their competitors were excluded due to their higher cost and lower efficacy. The ICER was then calculated using the following formula, and the strategy with the lowest cost-effectiveness ratio (C/E) was selected as optimal [14].

$$ \frac{C}{E}=\frac{\varDelta C}{\varDelta E}=\frac{Cost \left(evaluated\right)-Cost \left(standard\right)}{Effectiveness \left(evaluated\right)-Effectiveness\left(standard\right)}$$

The cost attributable to each branch of the decision tree was calculated by adding the costs associated with the clinical consultation, PCR and genotyping, follow up visits and the time taken for diagnostic testing, supervision and validation by both the specialist technician and the attending physician; this was multiplied in each case by the probability associated with each branch. The cost of additional visits by the specialist in charge were imputed in the case of false negatives. The costs attributable to healthcare employee hours invested and the cost associated with the medical consultation were obtained from official data published by the Conselleria de Sanitat [15] and are described in Table 1.

**Table 1:** Patient health care-associated costs during lymphogranuloma venereum diagnosis

Description	Unit	Cost (€)
Specialized Care	1 visit	50
Microbiology faculty work	1 h	22
Lab technician work	1 h	11
Costs incurred by L1-L3 *C. trachomatis* biovar detection	1 test	25
**Results classification**	**Strategy**	**Cost (€)**
True positive	Clinical strategy	100
False positive		100
True negative		50
False negative		150
True positive	Molecular strategy	192,5
False positive		-
True negative		142,5
False negative		242,5

### Sensitivity analysis

We used the deterministic threshold value method for sensitivity analysis: this involves arbitrary substitution of the probabilities defining the model within logical ranges to evaluate the influence of these changes on the decision taken, and thus find the critical value that would change the direction of the decision [16, 17]. To do this, we first increased the proportion of symptomatic patients gradually from 60 to 99%, then in a second analysis we increased the number of asymptomatic patients from 20 to 50%.

## Results

Of the 136 patients who met the inclusion criteria, samples were available in only 85, of which 14 corresponded to MSM co-infected with *Neisseria gonorrhoeae*, 25 to seropositive patients, 31 to patients included in PreP and 15 to MSM infected with CT but without another associated risk factors. The samples (one per patient) were stratified as follows: 35 urethral exudates, 31 from symptomatic patients, of which only 4 (13%) belonged to genogroups L1–L3 (all symptomatic). The remaining 50 samples were rectal exudates, 21 of which corresponded to symptomatic patients. In total, 25 of the 50 exudates belonged to the L1–L3 genogroup (50%), a significantly higher proportion (*p* < 0.001) than was observed in urethral exudates, and 17 (68%) of these 25 were from symptomatic patients. No cases of LGV were detected in the patient subgroup without associated risk factors (Table 2**).**

**Table 2:** Clinical-demographic features, sample type and genotyping results for C. trachomatis

Cohorts	LGV Result	Urethral exudate (n)	Rectal exudate (n)
No additional risk factors (*n* = 15)	POS	0	0
	NEG	14	1
Coinfected with CT and NG (*n* = 14)	POS	0	1
	NEG	12	1
HIV positive (*n* = 25)	POS	1	14
	NEG	4	6
PrEP (*n* = 31)	POS	3	10
	NEG	1	17

LGV: Lymphogranuloma venereum; CT. *Chlamydia trachomatis;* NG: *Neisseria gonorrhoeae;* HIV; Human immunodeficiency virus; PrEP; Pre-exposure Prophylaxis Program;

### Economic evaluation

The number of true positives (VP), false positives (FP), true negatives (VN) and false negatives (FN) for each of the four strategies is shown in Table 3.

**Table 3:** Patient characterization by diagnostic strategy used

Diagnostic strategy	True positive	True negative	False positive	False negative
I Syndromic	17	52	4	12
II MB- All rectal exudates with CT positive result	25	56	0	4
III MB- All rectal and urethral exudates of symptomatic patients with a CT positive result	21	56	0	8
IV All patients with a CT positive result	29	56	0	0

CT. *Chlamydia trachomatis*; MB: Molecular biology

In the clinical arms, the cost associated with TP and FP was 100 euros, corresponding to diagnostic and follow-up visits. The TN cost 50 euros (diagnostic visit only), and the FN totaled 150 euros (added appointment due to diagnostic error). In the arms with genotypic characterization, the TP corresponded to 192.5 euros (150 euros for medical visits, 17.5 euros for laboratory personnel hours and 25 euros for genotypic characterization reagents). The TN amounted to 142.5 euros for the two visits, laboratory time and reagents used for genotypic characterization. Finally, the FN cost 242.5 euros, resulting from the clinical visits, laboratory personnel costs, genotypic reagents and additional follow-up visit associated with diagnostic error (Table 1).

**Fig. 1:**
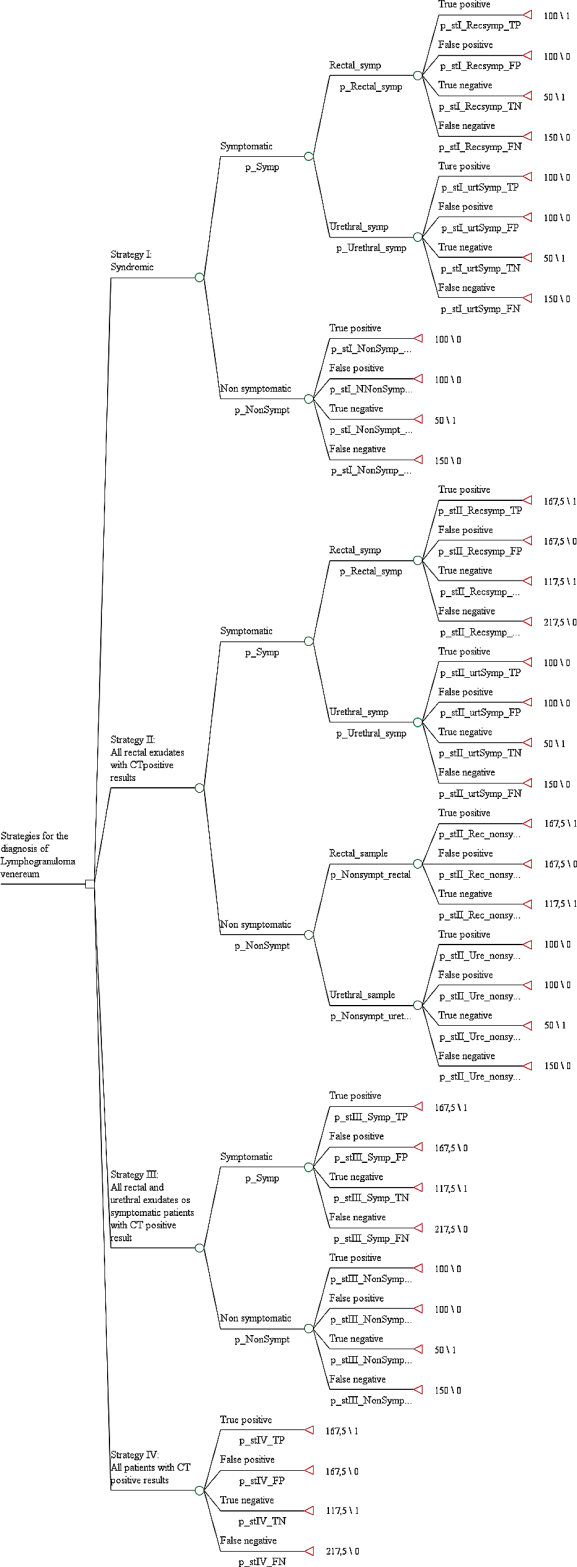
Decision tree for the selection of the most cost-effective strategy for LGV diagnostic

Regarding the decision tree (Fig. 1), the average cost per patient of the different strategies was 77.09, 123.84, 129.39 and 159.55 euros for strategies I, II, III and IV respectively, with effectiveness of 0.80, 0.95, 0.91 and 1.00. This gave an ICER of 311.67 for strategy II compared to strategy I, 412.3 for strategy IV compared to strategy I, and 714.2 for strategy IV compared to strategy II. Strategy III was dominated by its comparators (Supplementary Fig. 1).

### Sensitivity analysis

**Fig. 2:**
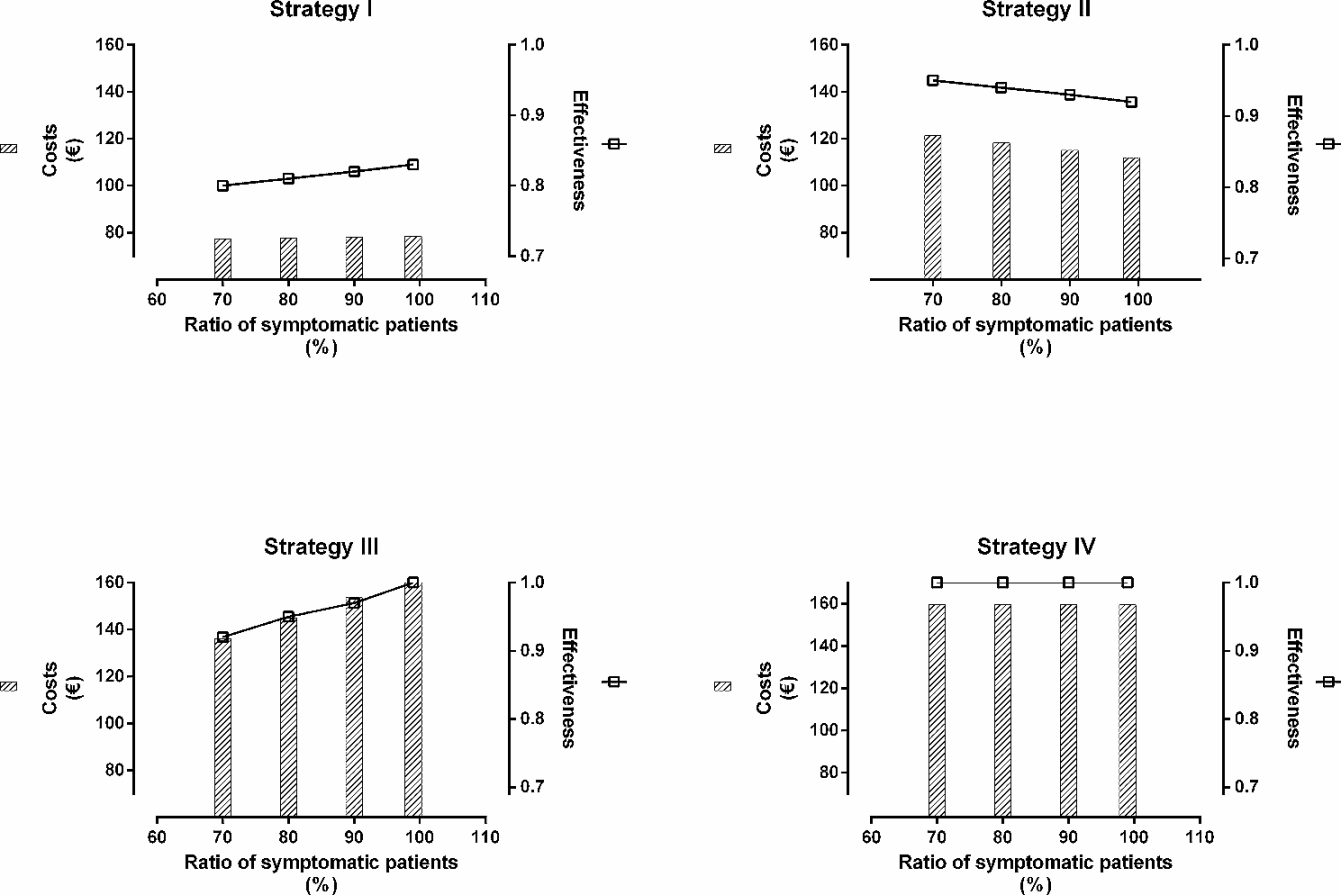
Influence of the ratio of symptomatic patients in the four strategies evaluated for the diagnosis of LGV

As shown in Fig. 2, the gradual increase in the proportion of symptomatic patients led to progressively higher effectiveness for strategies I and III, while the efficacy of strategy IV was maintained and that of strategy II decreased. The threshold ratio of symptomatic patients was 80%, from which point strategy number III ceased to be dominated, with ICER values of 312.23 € and 479.71€ for strategy II and strategy III respectively, compared to strategy I (Supplementary Table 1).

Studying the influence of asymptomatic patients on optimal strategy selection (Supplementary Fig. 2), the effectiveness of strategy II rose in parallel with an increased proportion of these patients. This was in contrast with other strategies: strategy I and III saw slightly reduced efficacy and strategy IV remained constant. Diagnostic costs decreased in strategies I and III compared to strategy II and IV, the former increasing slightly and the later remaining without changes.

Taking strategy I as a reference once more, the strategy with the lowest ICER at 20% asymptomatic patients was strategy II (312.46 euros). Thereafter, strategy II and IV become progressively closer, although without reaching a threshold value that would modify the meaning of our analysis (Suppementary Table 2).

## Discussion

LGV is an infectious pathology transmitted by the CT genogroups L1–L3, which had previously been associated with tropical and subtropical regions. Nonetheless, an outbreak of LGV detected in the Netherlands at the beginning of the century revealed the presence of a large number of asymptomatic patients, mainly in MSM groups [18]. In a previous work carried out in Portugal, Neves et al. [6] found the prevalence of L1–L3 among CT-positive patients to be close to 10%, but nearly 90% of these were seropositive MSM. Another study, carried out in France, observed in anorectal specimens that 20% of patients were L1–L3-positive [19]. In our study we found that 86.2% L1-L3 genotype CT belonged to anorectal exudates and 50% of all anorectal samples tested. The difference found with the abovementioned papers could be caused by the ratio of preselected patients. Taking into account only high risk VIH patients, our ratio of LGV-positive CT was 70%. This discrepancy also highlights the variability in prevalence of this pathology between population groups and underlines the importance of correct pre-analytical selection of patients to optimize resources [6].

Previous studies such as Vries [20] show that urogenital infections due to LGV are less frequent than rectal ones, with an estimated ratio of 1:15 for urogenital vs. rectal infections. In our case, the proportion observed was higher (1/6.25). This difference could be associated with the size of the population, given our limited sample size; however, an alternative form of transmission such as the oral-rectal route also stands out in this context. According to the previously mentioned study [8] as well as the European Guideline on the management of proctitis, proctocolitis and enteritis caused by sexually transmitted pathogens, the proportion of asymptomatic patients infected by LGV reaches between 25 and 30% of all cases of infection [20]. In our work, this value is very similar, reaching 27.5%, which further supports the use of strategies based on molecular biology such as II and IV compared to clinical strategies such as I or III.

Regarding the economic evaluation of the four diagnostic strategies, strategy II was the most cost-effective strategy in our cohort. Australian STI management guidelines [10] recommend testing for all MSM coinfected with another STI pathogen with related symptomatology, and MSM living with a seropositive partner even if asymptomatic provided they have a positive rectal exudate CT result [10]; in contrast, American guidelines support their diagnostic strategy on clinical suspicion and epidemiological information provided there is a positive CT result in a compatible anatomical site [11]. The 2021 European guidelines [6] recommend performing genotyping of all CT-positive anorectal samples from MSM patients and considering genotyping in patients with signs of proctitis/proctocolitis, femoral or inguinal lymphadenopathy or bubo, and/or history of genital ulcers, with priority given to patients with associated risk factors such as PrEP or HIV positive [4]. 

To our knowledge, no economic evaluation has been published which elucidates the most cost-effective strategy from among different possible alternatives. In Europe, where LGV prevalence among heterosexual patients is very low [4], initial diagnosis via patient screening is fundamental from an efficiency point of view. On the other hand, given the substantial proportion of asymptomatic patients, anorectal symptomatology is not in itself an adequate screening criterion. With the incidence detected in our healthcare area, therefore, the strategy that analyzes all CT positive rectal samples in MSM is the most cost-effective one and therefore we have adopted in our heathcare area. As the sensitivity study shows, an increased proportion of symptomatic patients improves the effectiveness of the test and therefore decreases the ICER ratio of strategies based on clinical diagnosis, or mixed strategies such as strategy III. However, there is a large body of evidence pointing to a high proportion of asymptomatic patients. In fact, this number is likely to increase as the proportion of the population undiagnosed for this pathology is reduced. Finally, the choice of less restrictive criteria, with the consequent reduction of the pretest effect, tends to select the strategy with highest sensitivity as the most profitable one, although we were unable to establish a threshold value that would select strategy IV, based entirely on molecular biology.

Our work has several limitations, the first of which is the small sample size, although this weakness was overcome by the sensitivity analysis. Second, in the cohort of patients using PreP, although all patients included in the program were screened in different anatomical locations, difficulties in control and follow-up during their stay in the program contributed to a lack of sufficient samples from this group. Third, no other studies reporting economic data associated with LGV diagnosis were found for comparison with our results. Fourth, the costs attributed to each branch were estimates, since a micro-costing strategy, which would reflect the expenditure specific to each strategy more accurately, was beyond the scope of this study. Likewise, without access to antibiotic prescription data we were unable to include treatment-associated costs in the economic study. Finally, both costs and efficacy were obtained with in-house experimental data in a specific cohort. This could affect the external validity of the results.

## Conclusions

Adequate patient selection enables us to optimize diagnostic strategies. The most cost-effective strategy in our setting was based on genotyping all rectal exudates of at-risk patients with a positive result for CT by PCR (Strategy II).

Less restrictive selection criteria for patient prescreening enables the most sensitive diagnostic approach (genotyping of all rectal samples or genotyping all CT-positive samples) to be the most cost-effective one.

### Electronic supplementary material

Below is the link to the electronic supplementary material.

Supplementary Material 1

Supplementary Material 2

Supplementary Material 3

Supplementary Material 4

## Data Availability

The datasets generated during and/or analyzed during the current study are available from the corresponding author on reasonable request.
