# Supplementary material for: Genotypic study of Chlamydia trachomatis for lymphogranuloma venereum diagnosis in rectal specimens from men who have sex with men: a cost-effectiveness analysis
Source: BMC Infect Dis. 2024 Mar 7;24:298. doi: 10.1186/s12879-024-09185-4 (PMC10918947; doi:10.1186/s12879-024-09185-4)
Supplement: Supplementary file 1 — Supplementary Material 1 [file 12879_2024_9185_MOESM1_ESM.docx]

| **Supplementary table 1. Increasing percentage of symptomatic patients** | | | | |
| --- | --- | --- | --- | --- |
| Ratio of symptomatic patients | Strategy number | Cost  (€) | Efficacy | ICER |
| 0.60 | I | 77.01 | 0.79 |  |
|  | II | 124.56 | 0.95 | 297.19 |
|  | III | 127.38 | 0.90 | 457.91 |
|  | IV | 159.55 | 1.00 | 393.05 |
| 0.7 | I | 77.35 | 0.80 |  |
|  | II | 121.42 | 0.95 | 293.80 |
|  | III | 136.11 | 0.92 | 489.67 |
|  | IV | 159.55 | 1.00 | 411.00 |
| 0.8 | I | 77.68 | 0.81 |  |
|  | II | 118.27 | 0.94 | 312.23 |
|  | III | 144.84 | 0.95 | 479.71 |
|  | IV | 159.55 | 1.00 | 430.89 |
| 0.9 | I | 78.02 | 0.82 |  |
|  | II | 115.12 | 0.93 | 337.27 |
|  | III | 153.57 | 0.97 | 503.67 |
|  | IV | 134.55 | 1.00 | 314.06 |
| 1.0 | I | 78.35 | 0.83 |  |
|  | II | 111.97 | 0.92 | 373.56 |
|  | III | 162.30 | 1.00 | 493.82 |
|  | IV | 159.55 | 1.00 | 477.65 |
| ICER: Incremental cost-effectiveness ratio | | | | |
