# Supplementary material for: Genotypic study of Chlamydia trachomatis for lymphogranuloma venereum diagnosis in rectal specimens from men who have sex with men: a cost-effectiveness analysis
Source: BMC Infect Dis. 2024 Mar 7;24:298. doi: 10.1186/s12879-024-09185-4 (PMC10918947; doi:10.1186/s12879-024-09185-4)
Supplement: Supplementary file 2 — Supplementary Material 2 [file 12879_2024_9185_MOESM2_ESM.docx]

| **Supplementary table 2.** **Increasing numbers of asymptomatic patients.** | | | | |
| --- | --- | --- | --- | --- |
| Ratio of asymptomatic patients | Strategy number | Cost  (€) | Efficacy | ICER |
| 0.2 | I | 77.68 | 0.81 |  |
|  | II | 118.3 | 0.94 | 312.46 |
|  | III | 144.84 | 0.95 | 479.71 |
|  | IV | 159.55 | 1 | 430.89 |
| 0.3 | I | 77.3 | 0.80 |  |
|  | II | 121.4 | 0.95 | 294.00 |
|  | III | 136.1 | 0.92 | 490.00 |
|  | IV | 159.55 | 1 | 411.25 |
| 0.4 | I | 77.0 | 0.80 |  |
|  | II | 124.6 | 0.95 | 317.33 |
|  | III | 127.4 | 0.90 | 504.00 |
|  | IV | 159.55 | 1 | 412.75 |
| 0.5 | I | 76.7 | 0.79 |  |
|  | II | 127.7 | 0.96 | 300.00 |
|  | III | 118.6 | 0.87 | 523.75 |
|  | IV | 159.55 | 1 | 394.52 |
| ICER: Incremental cost-effectiveness ratio | | | | |
